# Diabetes Technology: Markers, Monitoring, Assessment, and Control of Blood Glucose Fluctuations in Diabetes

**DOI:** 10.6064/2012/283821

**Published:** 2012-10-17

**Authors:** Boris P. Kovatchev

**Affiliations:** Department of Psychiatry and Neurobehavioral Sciences, Department of Systems and Information Engineering, Center for Diabetes Technology, and University of Virginia Health System, University of Virginia, P.O. Box 400888, Charlottesville, VA 22908, USA

## Abstract

People with diabetes face a life-long optimization problem: to maintain strict glycemic control without increasing their risk for hypoglycemia. Since the discovery of insulin in 1921, the external regulation of diabetes by engineering means has became a hallmark of this optimization. Diabetes technology has progressed remarkably over the past 50 years—a progress that includes the development of markers for diabetes control, sophisticated monitoring techniques, mathematical models, assessment procedures, and control algorithms. Continuous glucose monitoring (CGM) was introduced in 1999 and has evolved from means for retroactive review of blood glucose profiles to versatile reliable devices, which monitor the course of glucose fluctuations in real time and provide interactive feedback to the patient. Technology integrating CGM with insulin pumps is now available, opening the field for automated closed-loop control, known as the artificial pancreas. Following a number of in-clinic trials, the quest for a wearable ambulatory artificial pancreas is under way, with a first prototype tested in outpatient setting during the past year. This paper discusses key milestones of diabetes technology development, focusing on the progress in the past 10 years and on the artificial pancreas—still not a cure, but arguably the most promising treatment of diabetes to date.

## 1. Introduction

In health, glucose metabolism is tightly controlled by a hormonal network including the gut, the liver, the pancreas, and the brain to ensure stable fasting blood glucose (BG) levels and transient postprandial glucose fluctuations. In diabetes, this network control is disrupted by deficiency or absence of insulin secretion and/or insulin resistance, which has to be compensated by technological means. Generally, people with diabetes are classified into type 1 and the much more prevalent type 2 diabetes accounting for 90–95% of all cases. Type 1 diabetes is characterized by absolute deficiency of insulin secretion resulting from autoimmune response targeting the *β*-cells of the pancreas; type 2 diabetes is triggered by a combination of resistance to insulin and insufficient *β*-cell function [1]. 

For the 1,900 years following the clinical introduction of the term *diabetes* (Aretaeus the Cappadocian, 1st Century AD) diet was the only, albeit unsuccessful, treatment. In the 19th century, it was understood that diabetes is a complex of disorders characterized by a common final element of hyperglycemia (elevated blood sugar levels). With the discovery of insulin in 1921 by Frederick Banting at the University of Toronto, diabetes, particularly type 1, was no longer a death sentence. For this breakthrough, Banting and John Macleod were awarded the Nobel Prize in physiology or medicine in 1923. To recognize the contributions of their colleagues, Banting shared his prize with Charles Best and Macleod shared his with J. B. Collip. Insulin injections became the standard treatment for type 1 diabetes and for many people with type 2 diabetes. The field of diabetes technology was born. 

**Figure 1:**
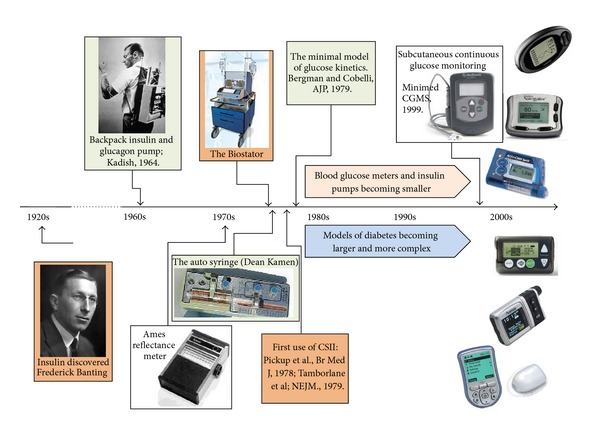
The Diabetes technology timeline from the discovery of insulin to the introduction of continuous glucose monitoring.

Forty years after the discovery of insulin—in 1963—an insulin pump delivering insulin and glucagon (to counteract hypoglycemia) was designed by Kadish [2]. In 1969, the first portable blood glucose meter—the Ames Reflectance meter—was manufactured. The first commercial subcutaneous insulin pump—the AutoSyringe—was introduced by Dean Kamen in the 1970s, and by the end of the decade the first trials of continuous subcutaneous insulin infusion (CSII) were reported by Pickup et al. in England [3] and Tamborlane et al. in the USA [4], showing the feasibility of this minimally-invasive mode of insulin replacement. The next logical step was automated insulin delivery controlled by a mathematical algorithm —a method that became known as closed-loop control, or the “artificial pancreas.” The artificial pancreas idea can be traced back to the 1970s when the possibility for external blood glucose regulation was established by studies using intravenous (i.v.) glucose measurement and i.v. infusion of glucose and insulin. Five teams reported i.v. closed-loop control results between 1974 and 1978: Albisser et al. [5], Pfeiffer et al. [6], Mirouze et al. [7], Kraegen et al. [8], and Shichiri et al. [9]. In 1977, one of these designs [6] resulted in the first commercial device—the Biostator [10]—a large (refrigerator-sized) device that has been used extensively for glucose-control research (Figure 1). Systems such as the Biostator have been employed in hospital settings to maintain normoglycemia using negative (via insulin) and positive (via glucose or glucagon) control [11–13]. A review of methods for i.v. glucose control can be found in [14].

In 1979, another key element of the closed-loop control—the Minimal Model of Glucose Kinetics—was introduced by Bergman and Cobelli [15]. This, and subsequent mathematical models, serves as the “brain” behind the majority of control algorithms used in contemporary artificial pancreas systems. Detailed description of the major early algorithm designs can be found in [16–19]. More work followed, spanning a range of control techniques powered by physiologic modeling and computer simulation [20–23]. 

The final critical technology leap enabling minimally invasive closed-loop designs was made at the turn of the century with the introduction of continuous glucose monitoring (CGM, [24–26])—an event that started the ongoing quest for wearable artificial pancreas. Figure 1 depicts the timeline of these events and the acceleration of diabetes technology development in the past two decades

## 2. Markers of Average Glycemia and Blood Glucose (BG) Fluctuations

### 2.1. Hemoglobin A1c (HbA1c)

In the early 1990s the landmark Diabetes Control and Complications Trial (DCCT, [27–29]) and the Stockholm Diabetes Intervention study [30] clearly indicated that intensive insulin treatment can reduce the long-term complications of type 1 diabetes. In 1998 the UK Prospective Diabetes Study group established that intensive treatment with insulin or with oral medications to maintain nearly normal levels of glycemia markedly reduces chronic complications in type 2 diabetes as well [31]. HbA1c was identified as the primary marker of long-term average glucose control [32, 33] and still remains the gold-standard assay reflecting average glycemia widely accepted in research as a primary outcome for virtually all studies of diabetes treatment and in the clinical practice as primary feedback to the patient and the physician and a base for treatment optimization.

### 2.2. Risk for Hypoglycemia

However, the DCCT also showed that intensive treatment of diabetes can also increase the risk for severe hypoglycemia (low blood glucose that could result in stupor, unconsciousness, and even death [34]. Indeed, HbA1c has repeatedly been proven to be an ineffective assessment of patients' risk for hypoglycemia. The DCCT concluded that only about 8% of severe hypoglycemic episodes could be predicted from known variables, including HbA1c [34]; later this prediction was improved to 18% by a structural equation model using history of severe hypoglycemia, awareness, and autonomic symptom score [35]. In subsequent studies, HbA1c has never been significantly associated with severe hypoglycemia [36–39]. Nevertheless, the physiological mechanisms of hypoglycemia were well established by a number of studies that have investigated the relationships between intensive therapy, hypoglycemia unawareness, and impaired counterregulation [40–43] and concluded that recurrent hypoglycemia spirals into a “vicious cycle” known as hypoglycemia-associated autonomic failure (HAAF, [44]) are observed primarily in type 1 and also in type 2 diabetes [45]. The acute risk for hypoglycemia was attributed to impairments in the systemic reaction to falling BG levels: in health, falling BG concentration triggers a sequence of responses, beginning with attenuation of endogenous insulin production, followed by increase in glucagon and epinephrine and, if BG concentration falls further, resulting in autonomic symptoms and/or neuroglycopenia; in type 1 diabetes, and to some extent in type 2 diabetes, these defense mechanisms are impaired [46–48]. As a result, hypoglycemia was identified as the primary barrier to optimal diabetes control [49, 50]. The clinical optimization problem of diabetes was therefore clearly formulated: reduce average glycemia and exposure to high blood glucose levels (thereby HbA1c), while preventing hypoglycemia.

### 2.3. Glucose Variability (GV)

Blood glucose variability is the primary challenge to the success of this optimization, and is typically at the root of clinicians' inability to safely achieve near-normal average glycemia, as reflected by HbA1c [51]. Indeed, in addition to establishing HbA1c as the gold standard for average glycemic control, the DCCT concluded that “HbA1c is not the most complete expression of the degree of glycemia. Other features of diabetic glucose control, which are not reflected by HbA1c, may add to, or modify the risk of complications. For example, the risk of complications may be highly dependent on the extent of postprandial glycemic excursions” [28]. As noted above, while target HbA1c values of 7% or less result in decreased risk of vascular complications [27, 29, 31, 33], the risk for severe hypoglycemia increases with tightening glycemic control [34, 48]. At the high end of the BG scale, a number of studies have implicated postprandial BG fluctuation as an independent risk factor for diabetes complications [52, 53], particularly cardiovascular disease [54–57]. Thus, a strategy for achieving optimal glucose control can only be successful if it reduces GV. This is because bringing average glycemia down is only possible if GV is constrained, otherwise BG fluctuations would inevitably enter the range of hypoglycemia (see, [51, Figure  4]). 

### 2.4. Behavioral Triggers of Glucose Variability

Formulated from an engineering point of view, the control of diabetes is driven by routine self-treatment behaviors which may occasionally evolve into hypo- or hyperglycemia-triggering events, for example, insulin mistiming, bolus/basal imbalance, missed meal, or excessive exercise. A formal mathematical description of this process and its potential to destabilize the system was given by the Stochastic Model of Self-Regulation Behavior which provided a probabilistic interpretation of the event sequence: internal condition **→** perception/awareness **→** appraisal **→** self-regulation decision [58–61]. The parameters of this process are individual, contingent on behavioral interpretation, for example, on a person's ability to control his/her BG within optimal limits. The effect of this process is mediated by the specifics of a person's metabolic system, such as rate of glucose appearance in the blood, or insulin sensitivity. The net result from this biobehavioral interplay is a certain degree of glucose variability, which in turn could provide feedback to the person regarding the effectiveness of his/her glycemic control and could prompt corrective actions if needed, for example, adjustment of insulin timing or bolus/basal ratio. 

### 2.5. Physiological Mechanisms of GV

Once triggered, the progression and the extent of glycemic excursions depend on individual parameters of insulin transport, insulin sensitivity, and counterregulatory response. Technologies utilizing subcutaneous insulin injection (e.g., CSII, artificial pancreas) rely on the transport of s.c.-injected insulin into the circulation. The duration of this transport varies from person to person and is a major mechanism of postprandial GV because of the introduced delays—a postprandial excursion has time to develop due to insulin deficiency (relative to health) in its initial stages, even if a meal bolus is given on time. Thus, the modeling and the formal description of s.c. insulin transport is important for the effectiveness of modern diabetes control strategies [62, 63]. After entering the circulation, the action of insulin is determined by the dynamics of insulin-mediated glucose utilization—a process that has been mathematically characterized by Bergman and Cobelli's classic Minimal Model, which introduced the mathematical formulation of *insulin sensitivity* [15], a key metabolic parameter that has been the subject of investigation of a number of subsequent studies [64–70]. It is now well known that insulin sensitivity is enhanced by exercise [71–74]; methods exist for quantitative assessment of insulin sensitivity in laboratory [64] and in outpatient settings [67], including methods for assessment during physical activity [75]. The processes of gastric emptying and glucose appearance in the blood are similarly well-quantified [76, 77]. It is apparent that a major source of GV is rapid onset of hyperglycemia due to consumption of “high glycemic index” foods, especially in large quantity. For example, foods with simple carbohydrate and high fat (classically pizza) present challenges to technology and optimal therapy by resulting in sustained postprandial hyperglycemia. 

## 3. Monitoring of BG Fluctuations in Diabetes

### 3.1. The Frequency of BG Observation

Intuitively, the aggressiveness of glucose control in diabetes would depend on the frequency of glucose measurement. For example, if only the average glycemic state of a patient is available once every few months (as it would be with measurement of HbA1c alone), then control strategies could only target adjustment of long-term average glycemia, but would not be able to respond to daily or hourly variation in glucose level. Rapid BG changes would remain largely unnoticed, unless they led to acute complications, such as severe hypoglycemia or diabetic ketoacidosis. Thus, the frequency of glucose measurement determines, to a large extent, the aggressiveness of possible treatments.  Table 1 presents the frequency and the temporal resolution of commonly used glucose assessment techniques. Generally, HbA1c reflects long-term (over 2-3 months) blood glucose average; thus the temporal resolution of HbA1c is limited to reflect slow changes in average glycemia. Self-monitoring of blood glucose (SMBG) is a standard practice including several (e.g., 2–5) BG readings per day. Thus, the temporal resolution of SMBG allows for assessment of daily BG profiles, or weekly trends. With the advent of CGM, it is now well accepted that BG fluctuations are a process in time, which has two principal components: risk, associated with the amplitude (variability) of BG changes, and time indicating the rate of event progression. Contemporary CGM devices are capable of producing BG determinations every 5–10 minutes, which provides vast amounts of data with high temporal resolution and allows for detailed monitoring of glucose fluctuations on a temporal scale of minutes—a frequency that enables closed-loop control. 

### 3.2. Self-Monitoring of Blood Glucose

Contemporary home BG meters offer convenient means for frequent and accurate BG determinations through self-monitoring. Most devices are capable of storing BG readings (typically over 150 readings) and have interfaces to download these readings into a computer. The meters are usually accompanied by software that has capabilities for basic data analyses (e.g., calculation of mean BG, estimates of the average BG over the previous two weeks, percentages in target, hypoglycemic and hyperglycemic zones, etc.) and log of the data, and graphical representation (e.g., histograms, pie charts) [78–81]. Analytical methods based on SMBG data are discussed in the next section. 

### 3.3. Continuous Glucose Monitoring

Since the advent of CGM technology 10 years ago [25–27], significant progress has been made towards versatile and reliable CGM devices that not only monitor the entire course of BG day and night, but also provide feedback to the patient, such as alarms when BG reaches preset low or high levels. A number of studies have documented the benefits of CGM [82–85] and charted guidelines for clinical use and its future as a precursor to closed-loop control [86–89]. However, while CGM has the potential to revolutionize the control of diabetes, it also generates data streams that are both voluminous and complex. The utilization of such data requires an understanding of the physical, biochemical, and mathematical principles and properties involved in this new technology. It is important to know that CGM devices measure glucose concentration in a different compartment—the interstitium. Interstitial glucose (IG) fluctuations are related to BG presumably via diffusion process [90–92]. To account for the gradient between BG and IG, CGM devices are calibrated with capillary glucose, which brings the typically lower IG concentration to BG levels. Successful calibration would adjust the amplitude of IG fluctuations with respect to BG, but would not eliminate the possible time lag due to BG-to-IG glucose transport and the sensor processing time (instrument delay). Because such a time lag could greatly influence the accuracy of CGM, a number of studies were dedicated to its investigation, yielding various results [93–96]. For example, it was hypothesized that if a glucose fall is due to peripheral glucose consumption the physiologic time lag would be negative, that is, fall in IG would precede fall in BG [90, 97]. In most studies IG lagged behind BG (most of the time) by 4–10 minutes, regardless of the direction of BG change [92, 93]. The formulation of the push-pull phenomenon offered reconciliation of these results and provided arguments for a more complex BG-IG relationship than a simple constant or directional time lag [96, 98]. In addition, errors from calibration, loss of sensitivity, and random noise confound CGM data [99]. Nevertheless, the accuracy of CGM is increasing and may be reaching a physiological limit for subcutaneous glucose monitoring [100–103]. 

In addition to presenting frequent data (e.g., every 5–10 minutes), CGM devices typically display directional trends and BG rate of change and are capable of alerting the patient of upcoming hypo- or hyperglycemia. These features are based on methods which predict blood glucose and generate alarms and warning messages. In the past several years these methods have rapidly evolved from a concept [104] to implementation in CGM devices, such as the Guardian RT and the MiniMed Paradigm REAL-Time System (Medtronic, Norhtridge, CA, USA) [105] and the Freestyle Navigator (Abbott Diabetes Care, Alameda, CA, USA) [106]. Alarms for particularly rapid rates of BG change (e.g., greater than 2 mg/dL/min) are available as well (Guardian RT and Dexcom Seven Plus, Dexcom, San Diego, CA, USA). Discussion of the methods for testing of the accuracy and the utility of such alarms has been initiated [106–108], and the next logical step—prevention of hypoglycemia via shutoff of the insulin pump—has been undertaken [109]. 

## 4. Assessment of BG Fluctuations in Diabetes

As presented in Table 1, different frequencies of BG monitoring provide data for different types of analytical techniques assessing the glycemic state, or the BG dynamics, of a person with diabetes. A brief account of analytical methods applicable to SMBG and/or CGM data is given below. Some of these methods, such as the Risk Analysis of BG data, have entered the design of closed-loop control systems preventing hypoglycemia [110].

### 4.1. SMBG-Based Analytical Methods

The computation of mean glucose values from SMBG data is typically used as a descriptor of overall glycemic control. Computing pre- and postmeal averages and their difference can serve as an indication of the effectiveness of premeal bolus timing and amount. Similarly, the percentages of SMBG readings within, below, or above preset target limits would serve as indication of the general behavior of BG fluctuations. The suggested limits are 70 and 180 mg/dL (3.9–10 mmol/l), which create three suggested by the DCCT and commonly accepted bands: hypoglycemia (BG ≤ 70 mg/dL); normoglycemia (70 mg/dL < BG ≤ 180 mg/dL); hyperglycemia (BG > 180 mg/dL) [1]. Percentage of time within additional bands can be computed as well to emphasize the frequency of extreme glucose excursions. Computing standard deviation (SD) as a measure of glucose variability is not recommended because the BG measurement scale is highly asymmetric, the hypoglycemic range is numerically narrower than the hyperglycemic range, and the distribution of the glucose values of an individual is typically quite skewed [111]. Therefore, SD would be predominantly influenced by hyperglycemic excursions and would not be sensitive to hypoglycemia. It is also possible for confidence intervals based on SD to assume unrealistic negative values. Thus, as a standard measure of GV we would suggest reporting interquartile range (IQR), which is suitable for asymmetric distributions. Several diabetes-specific metrics are also available to serve the analysis of SMBG data, including the mean amplitude of glucose excursions (MAGE, [112]), the *M*-value [113], the lability index [114], and the low and high blood glucose indices (LBGI, HBGI) which reflect the risks associated with hypo- and hyperglycemia, respectively [37, 115]. In a series of studies we have shown that specific risk analysis of SMBG data could also capture long-term trends towards increased risk for hypoglycemia [36–38] and could identify 24-hour periods of increased risk for hypoglycemia [39, 116].

### 4.2. Risk Analysis of BG Data

To provide a flavor for the analytical techniques used for SMBG, CGM, and closed-loop control data, we will present a bit more detail on the concept for risk analysis of BG data [117]. The risk analysis steps are as follows. 

#### 4.2.1. Symmetrization of the BG Scale

A nonlinear transformation is applied to the BG measurements scale to map the entire BG range (20 to 600 mg/dL, or 1.1 to 33.3 mmol/l) to a symmetric interval. This is needed because the distribution of BG values of a person with diabetes is asymmetric, typically skewed towards hyperglycemia. The BG value of 112.5 mg/dL (6.25 mmol/l) is mapped to zero, corresponding to zero risk for hypo- or hyperglycemia (we should note that this is not a normoglycemic or fasting value, which in health would be <100 mg/dL; it is zero-risk value pertinent to diabetes). The analytical form of this transformation is *f*(BG) = *γ* · (ln⁡(BG)^*α*^ − *β*), where the parameters are estimated as *α* = 1.084,  *β* = 5.381, and *γ* = 1.509, if BG is measured in mg/dL and *α* = 1.026, *β* = 1.861, and *γ* = 1.794, if BG is measured in mmol/l [111]. 

#### 4.2.2. Assignment of a Risk Value to Each BG Reading

A quadratic risk function is defined as by the formula *r*(BG) = 10 · *f*(BG)^2^. The function *r*(BG) ranges from 0 to 100. Its minimum value is achieved at BG = 112.5 mg/dL, a safe euglycemic BG reading, while its maximum is reached at the extreme ends of the BG scale. Thus, *r*(BG) can be interpreted as a measure of the risk associated with a certain BG level. The left branch of this parabola identifies the risk of hypoglycemia, while the right branch identifies the risk of hyperglycemia. 

#### 4.2.3. Computing Measures of Risk for Hypoglycemia, Hyperglycemia, and Glucose Variability

Let *x*_1_, *x*_2_,…, *x*_*n*_ be a series of *n* BG readings, and let *rl*(BG) = *r*(BG) if *f*(BG) < 0 and 0 otherwise; *rh*(BG) = *r*(BG) if *f*(BG) > 0 and 0 otherwise. Then the low and high blood glucose indices are computed as follows:

(1) $$\begin{array}{c} \text{LBGI}=\frac{1}{n}\sum_{i=1}^{n}rl{\left(x_{i}\right)}^{2}\;\;\text{HBGI}=\frac{1}{n}\sum_{i=1}^{n}rh{\left(x_{i}\right)}^{2}. \end{array}$$

Thus, the LBGI is a nonnegative quantity that increases when the number and/or extent of low BG readings increases and the HBGI is nonnegative quantity that increases when the number and/or extent of high BG readings increases. Based on this same technique, we also define the average daily risk range (ADRR), which is a measure of risks associated with overall glycemic variability [118]. In studies, the LBGI typically accounted for 40–55% of the variance of future significant hypoglycemia in the subsequent 3–6 months [36–38], which made it a potent predictor of hypoglycemia based on SMBG. The ADRR has been shown superior to traditional glucose variability measures in terms of risk assessment and prediction of extreme glycemic excursions [118]. Specifically, it has been demonstrated that classification of risk for hypoglycemia based on four ADRR categories low risk: ADRR < 20; low-moderate risk: 20 ≤ ADRR < 30; moderate-high risk: 30 ≤ ADRR < 40; and high risk: ADRR > 40, resulted in more than a sixfold increase in risk for hypoglycemia from the lowest to the highest risk category [118]. In addition, the low and high BG indices have been adapted to continuous monitoring data [119] and can be used in the same way as with SMBG to assess the risk for hypo- or hyperglycemia. 

### 4.3. CGM-Based Analytical Methods

While traditional risk [119] and variability [120] analyses are still applied to CGM data, the high temporal resolution of CGM brought about the possibility for use of sophisticated analytical methods assessing system (person) dynamics on the time scale of minutes. This necessitated the development of new technologies for data analysis and visualization that are not available for SMBG data. Analysis of the BG rate of change (measured in mg/dL/min) is a way to evaluate the dynamics of BG fluctuations on the time scale of minutes. The BG rate of change at *t*_*i*_—is computed as the ratio (BG(*t*_*i*_) − BG(*t*_*i*−1_))/(*t*_*i*_ − *t*_*i*−1_), where BG(*t*_*i*_) and BG(*t*_*i*−1_) are CGM readings taken at times *t*_*i*_ and *t*_*i*−1_, for example, minutes apart. Investigation of the frequency of glucose fluctuations showed that optimal evaluation of the BG rate of change would be achieved over time periods of 15 minutes [121], for example, Δ*t* = *t*_*i*_ − *t*_*i*−1_ = 15. A large variation of the BG rate of change indicates rapid and more pronounced BG fluctuations and therefore a less stable system. Thus, the standard deviation of the BG rate of change is a measure of stability of glucose fluctuation (we should note that, as opposed to the distribution of BG levels, the distribution of the BG rate of change is *symmetric* and therefore, using SD is statistically accurate [122]). The SD of BG rate of change has been introduced as a measure of stability computed from CGM data, and is known as CONGA of order 1. In general, CONGA122 of order *n* is computed as the standard deviation of CGM readings that are *n* hours apart, reflecting glucose stability over these time intervals [123]. 

Most important to the development of the artificial pancreas algorithms is a class of methods allowing the prediction of BG values ahead in time. These methods, typically based on time-series techniques, have been applied successfully in a number of studies [124–129]. In addition to time series, neural networks have been used for the prediction of glucose levels from CGM [130, 131]. Detailed reviews of CGM data analysis methods are presented in [122], including several graphs that could be used for the visualization of the rather complex CGM data sets and in [132] where a broad review of modeling, analytical, and control techniques for diabetes is provided. 

## 5. Control of BG Fluctuations in Diabetes

### 5.1. Intraperitoneal Insulin Delivery

As detailed in the Introduction, the artificial pancreas idea can be traced back to the early 70s, when external BG regulation in people with diabetes was achieved by i.v. glucose measurement and i.v. infusion of glucose and insulin. However, the intravenous route of closed-loop control remains cumbersome and unsuited for outpatient use. An alternative has been presented by implantable intraperitoneal (i.p.) systems employing i.v. sampling and i.p. insulin delivery [133–136]. The i.p. infusion route has several desirable characteristics: reproducibility of insulin absorption, quick time to peak and return to baseline of insulin action, near-physiological peripheral insulin levels, and restoration of glucagon response to hypoglycemia and exercise [133, 137–139]. However, while i.p. systems have achieved excellent BG control, their implementation still requires considerable surgery and is associated with significant cost. Nevertheless, the development of less invasive and cheaper implantable ports (e.g., DiaPort, Roche Diagnostics, Mannheim, Germany) may contribute to the future proliferation of i.p. insulin delivery [140–142].

### 5.2. The Subcutaneous Route to Closed-Loop Control

**Figure 2:**
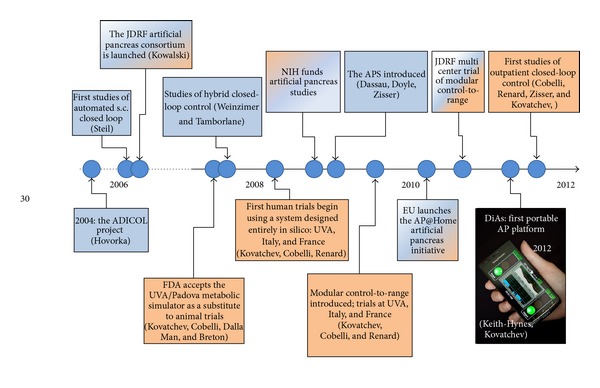
Timeline of the artificial pancreas developments in the last decade—theoretical work and a number of in-clinic studies leading to the first trials of wearable artificial pancreas device.

Following the progress of minimally invasive subcutaneous CGM, the next logical step was the development of s.c. closed-loop glucose control, which links a CGM device with CSII insulin pump. A key element of this combination was a control algorithm, which monitors BG fluctuations and the actions of the insulin pump, and computes insulin delivery rate every few minutes [143].  Figure 2 presents key milestones in the timeline of this development.

Following the pioneering work of Hovorka et al. [144, 145] and Steil et al. [146], in 2006, the Juvenile Diabetes Research Foundation (JDRF) initiated the Artificial Pancreas Project and funded several centers in the USA and Europe to carry closed-loop control research [147]. In 2008, the USA National Institutes of Health launched an artificial pancreas initiative, and in 2010, the European AP@Home consortium was established. By the end of the first decade of this century, the artificial pancreas became a global research topic engaging physicians and engineers in unprecedented collaboration [148, 149].

### 5.3. ***In Silico* Models of the Human Metabolic System**

A critical step towards accelerated clinical progress of the artificial pancreas was the development of sophisticated computer simulator of the human metabolic system allowing rapid *in silico *testing of closed-loop control algorithms. This simulation environment was based on the previously introduced Meal Model of glucose-insulin dynamics [76, 77] and was equipped with a “population” of *in silico* images of *N* = 300 “subjects” with type 1 diabetes, separated in three age groups: *N* = 100 simulated “children” below the age of 11; *N* = 100 “adolescents” 12–18 years old and *N* = 100 “adults.” The characteristics of these “subjects” (e.g., weight, daily insulin dose, carbohydrate ratio, etc.) were tailored to span a wide range of intersubject variability approximating the variability observed in people in vivo [150]. Simulation experiments allow any CGM device, any insulin pump, and any control algorithm to be linked in a closed-loop system *in silico*, prior to their use in clinical trials. With this technology, any meal and insulin delivery scenario can be pilot-tested very efficiently—a 24-hour period of closed-loop control is simulated in under 2 seconds. We need to emphasize, however, that good *in silico* performance of a control algorithm does not guarantee *in vivo* performance—it only helps test extreme situations and the stability of the algorithm, and rule out inefficient scenarios. Thus, computer simulation is only a prerequisite to, but not a substitute for, clinical trials. 

In January 2008, in an unprecedented decision, the USA Food and Drug Administration accepted this computer simulator as a substitute to animal trials for the testing of closed-loop control strategies. This opened the field for efficient and cost-effective *in silico* experiments leading directly to human studies. Only three months later, in April 2008, the first human trials began at the University of Virginia (USA), Montpellier (France), and Padova (Italy), using a control system designed entirely *in silico* [151]. 

### 5.4. Control System Designs

The first studies of Hovorka et al. [144, 145] and Steil et al. [146] outlined the two major types of closed-loop control algorithms now in use in artificial pancreas systems—model-predictive control (MPC, [145]) and proportional-integral-derivative (PID, [146]), respectively. By 2007, the blueprints of the contemporary controllers were in place, including run-to-run control [152–154] and linear MPC [155]. To date, the trials of subcutaneous closed-loop control systems have been using either PID [146, 156] or MPC [157–160], but MPC became the approach of choice targeted by recent research. There were two important reasons making MPC preferable: (i) PID is purely reactive, responding to changes in glucose level, while a properly tuned MPC allows for prediction of glucose dynamics and, as a result, for mitigation of the time delays inherent with subcutaneous glucose monitoring and subcutaneous insulin infusion [62, 63]; (ii) MPC allows for relatively straightforward personalizing of the control using patient-specific model parameters. In addition, MPC could have “learning” capabilities—it has been shown that a class of algorithms (known as run-to-run control) can “learn” specifics of patients' daily routine (e.g., timing of meals) and then optimize the response to a subsequent meal using this information or account for circadian fluctuation in insulin resistance (e.g., dawn phenomenon observed in some people) [149].

In 2008, a universal research platform—the APS—was introduced enabling automated communication between several CGM devices, insulin pumps, and control algorithms [161]. The APS was very instrumental for a number of inpatient trials of closed-loop control. A year later, a modular architecture was introduced, proposing standardization, sequential testing and clinical deployment of artificial pancreas components [162]. 

### 5.5. Inpatient Clinical Trials

Between 2008 and 2011, promising results were reported by several groups [156–160, 163–167]. Most of these studies pointed out the superiority of closed-loop control over standard CSII therapy in terms of (i) increased time within target glucose range (typically 3.9–10 mmol/l); (ii) reduced incidence of hypoglycemia and (iii) better overnight control. Two of these studies [159, 166] had state-of-the-art randomized cross-over design, but lacked automated data transfer—all CGM readings were transferred to the controller manually by the study personnel, and all insulin pump commands were entered manually as well. To distinguish the various degrees of automation in closed-loop studies, the notion of fully-integrated closed-loop control emerged, defined as having all of the following three components: (i) automated data transfer from the CGM to the controller, (ii) real-time control action, and (iii) automated command of the insulin pump. The first (and the largest to date) randomized cross-over study of fully-integrated closed-loop control was published in 2012 [168]. However, even this contemporary trial of fully automated CLC, which enrolled 38 patients with T1D at three centers and tested two different control algorithms achieving noteworthy glycemic control and prevention of hypoglycemia, did not leave the clinical setting. The technology used by this study was still based on a laptop computer wired to a CGM and an insulin pump, a system limiting the free movement of the study subjects and too cumbersome to be used beyond hospital confines. 

### 5.6. Wearable Outpatient Artificial Pancreas

The transition of closed-loop control to ambulatory use began in 2011 with the development of the Diabetes Assistant (DiAs)—the first wearable artificial pancreas platform based on a smart phone. The design characteristics of DiAs included the following:

- based on readily available, inexpensive, wearable hardware platform;
- computationally capable of running advanced closed-loop control algorithms;
- wirelessly connectable to CGM devices and insulin pumps;
- capable of broadband communication with a central location for remote monitoring and safety supervision of the participants in outpatient clinical trials.

In October 2011, the first two pilot trials of wearable outpatient artificial pancreas were performed simultaneously in Padova (Italy) and Montpellier (France) [169]. These 2-day trials allowed the refinement of a wearable system and enabled a subsequent multisite feasibility study of ambulatory artificial pancreas, which was completed recently at the Universities of Virginia, Padova, and Montpellier, and at the Sansum Diabetes Research institute, Santa Barbara, CA, USA. Results from this study are forthcoming.

## 6. Conclusions

Solving the optimization problem of diabetes requires replacement of insulin action through insulin injections or oral medications (applicable primarily to type 2 diabetes) which, until fully automated closed-loop control becomes available, would remain a process largely controlled by patient behavior. In engineering terms, BG fluctuations in diabetes result from the activity of a complex metabolic system perturbed by behavioral challenges. The frequency and extent of these challenges, and the ability of the person's metabolic system to absorb them, determines the quality of glycemic control. Along with HbA1c, the magnitude and speed of BG fluctuations, is the primary measurable marker of glucose control in diabetes. These same quantities—HbA1c and glucose variability—are also the principal feedback available to patients to assist with optimization of their diabetes control. 

In the past 30 years the technology for monitoring of blood glucose levels in diabetes has progressed from assessment of average glycemia via HbA1c once in several months, through daily SMBG, to minutely continuous glucose monitoring. The increasing temporal resolution of the monitoring technology enabled increasingly intensive diabetes treatment, from daily insulin injections or oral medication, through insulin pump therapy, to the artificial pancreas. This progress is accompanied by increasingly sophisticated analytical methods for retrieval of blood glucose data ranging from subjective interpretation of glucose values and straightforward summary statistics, through risk and variability analysis, to real-time closed-loop control algorithms based on complex models of the human metabolism.

It is therefore evident that the development of diabetes technology is accelerating exponentially. A primary catalyst of this acceleration is unprecedented interdisciplinary collaboration between physicians, chemists, engineers, and mathematicians. As a result, a wearable artificial pancreas suitable for outpatient use is now within reach. 

The primary engineering challenges to the widespread adoption of closed-loop control as a viable therapeutic option for diabetes include system connectivity, the accuracy of subcutaneous glucose sensing, and the speed of action of subcutaneously injected insulin. These challenges are well understood by those working in the field: wireless communication between CGM devices, insulin pumps, and closed-loop controllers are under development and testing, new generations of CGM device demonstrate superior accuracy and reliability, and new insulin analogs and methods for insulin delivery are being engineered to approximate as close as possible the action profile of endogenous insulin. It should be noted, however, that the signals available to a contemporary closed-loop control system are generally limited to CGM and insulin delivery data; user input about carbohydrate intake and physical activity could be available as well. In contrast, the endocrine pancreas receives direct and rapid control inputs from other nutrients (e.g., lipids and amino acids), adjacent cells (somatostatin from the delta cells and glucagon from alpha cells), incretins, and neural signals. Thus, while artificial closed-loop control is expected to be vastly superior to the diabetes control methods employed in the clinical practice today, it will continue to be imperfect when compared to the natural endocrine regulation of blood glucose. 

## Figures and Tables

**Table 1:** Frequency of available glucose monitoring technologies.

Measure	Temporal resolution	Reflects	Methods typically used to present and analyze the data
HbA1c	Months/years	Slow changes in average BG	Direct assay and review of values; group comparisons when treatments are evaluated

Self-monitoring of blood glucose (SMBG)	Days/weeks	Daily variation; weekly trends	Mean and standard deviation (SD), coefficient of variation (CV), Interquartile range (IQR); *M*-value (1965), MAGE (1970); lability index (2004); low/high BG (risk) Indices (1998); average daily risk range (ADRR, 2006).

Continuous glucose monitoring (CGM)	Minutes/hours	System dynamics, fluctuation, and periodicity	CONGA (2005); glucose rate of Change & CGM versions of low/high BG (risk) indices (2005); time series/dynamical system analysis

## References

[1] American Diabetes Association (2004). Diagnosis and classification of diabetes mellitus. *Diabetes Care*.

[2] Kadish AH (1964). Automation control of blood sugar—I. A servomechanism for glucose monitoring and control. *American Journal of Medical Electronics*.

[3] Pickup JC, Keen H, Parsons JA, Alberti KGMM (1978). Continuous subcutaneous insulin infusion: an approach to achieving normoglycaemia. *British Medical Journal*.

[4] Tamborlane WV, Sherwin RS, Genel M, Felig P (1979). Reduction to normal of plasma glucose in juvenile diabetes by subcutaneous administration of insulin with a portable infusion pump. *New England Journal of Medicine*.

[5] Albisser AM, Leibel BS, Ewart TG (1974). An artificial endocrine pancreas. *Diabetes*.

[6] Pfeiffer EF, Thum Ch., Clemens AH (1974). The artificial beta cell: a continuous control of blood sugar by external regulation of insulin infusion (glucose controlled insulin infusion system). *Hormone and Metabolic Research*.

[7] Mirouze J, Selam JL, Pham TC, Cavadore D (1977). Evaluation of exogenous insulin homoeostasis by the artifical pancreas in insulin dependent diabetes. *Diabetologia*.

[8] Kraegen EW, Campbell LV, Chia YO (1977). Control of blood glucose in diabetics using an artificial pancreas. *Australian and New Zealand Journal of Medicine*.

[9] Shichiri M, Kawamori R, Yamasaki Y, Inoue M, Shigeta Y, Abe H (1978). Computer algorithm for the artificial pancreatic beta cell. *Artificial Organs*.

[10] Clemens AH, Chang PH, Myers RW (1977). The development of Biostator, a Glucose Controlled Insulin Infusion System (GCIIS). *Hormone and Metabolic Research*.

[11] Marliss EB, Murray FT, Stokes EF (1977). Normalization of glycemia in diabetics during meals with insulin and glucagon delivery by the artificial pancreas. *Diabetes*.

[12] Santiago JV, Clemens AH, Clarke WL, Kipnis DM (1979). Closed-loop and open-loop devices for blood glucose control in normal and diabetic subjects. *Diabetes*.

[13] Fischer U, Jutzi E, Freyse EJ, Salzsieder E (1978). Derivation and experimental proof of a new algorithm for the artificial B-cell based on the individual analysis of the physiological insulin-glucose relationship. *Endokrinologie*.

[14] Parker RS, Doyle FJ, Peppas NA (2001). The intravenous route to blood glucose control: a review of control algorithms for noninvasive monitoring and regulation in type I diabetic patients. *IEEE Engineering in Medicine and Biology Magazine*.

[15] Bergman RN, Ider YZ, Bowden CR, Cobelli C (1979). Quantitative estimation of insulin sensitivity. *The American Journal of Physiology*.

[16] Broekhuyse HM, Nelson JD, Zinman B, Albisser AM (1981). Comparison of algorithms for the closed-loop control of blood glucose using the artificial beta cell. *IEEE Transactions on Biomedical Engineering*.

[17] Clemens AH (1979). Feedback control dynamics for glucose controlled insulin infusion system. *Medical Progress through Technology*.

[18] Cobelli C, Ruggeri A (1983). Evaluation of portal/peripheral route and of algorithms for insulin delivery in the closed-loop control of glucose in diabetes: a modeling study. *IEEE Transactions on Biomedical Engineering*.

[19] Salzsieder E, Albrecht G, Fischer U, Freyse EJ (1985). Kinetic modeling of the glucoregulatory system to improve insulin therapy. *IEEE Transactions on Biomedical Engineering*.

[20] Brunetti P, Cobelli C, Cruciani P (1993). A simulation study on a self-tuning portable controller of blood glucose. *International Journal of Artificial Organs*.

[21] Fischer U, Schenk W, Salzsieder E, Albrecht G, Abel P, Freyse EJ (1987). Does physiological blood glucose control require an adaptive control strategy?. *IEEE Transactions on Biomedical Engineering*.

[22] Sorensen JT (1985). *A physiologic model of glucose metabolism in man and its use to design and assess improved insulin therapies for diabetes [Ph.D. dissertation]*.

[23] Parker RS, Doyle FJ, Peppas NA (1999). A model-based algorithm for blood glucose control in type I diabetic patients. *IEEE Transactions on Biomedical Engineering*.

[24] Mastrototaro JJ (2000). The MiniMed continuous glucose monitoring System. *Diabetes Technology and Therapeutics*.

[25] Bode BW (2000). Clinical utility of the continuous glucose monitoring system. *Diabetes Technology and Therapeutics*.

[26] Feldman B, Brazg R, Schwartz S, Weinstein R (2003). A continuous glucose sensor based on wired enzyme technology—results from a 3-day trial in patients with type 1 diabetes. *Diabetes Technology and Therapeutics*.

[27] The Diabetes Control and Complications Trial Research Group (1993). The effect of intensive treatment of diabetes on the development and progression of long-term complications of insulin-dependent diabetes mellitus. *New England Journal of Medicine*.

[28] The Diabetes Control and Complications Trial Research Group (1995). The relationship of glycemic exposure (HbA1c) to the risk of development and progression of retinopathy in the Diabetes Control and Complications Trial. *Diabetes*.

[29] Lachin JM, Genuth S, Nathan DM, Zinman B, Rutledge BN (2008). Effect of glycemic exposure on the risk of microvascular complications in the diabetes control and complications trial-revisited. *Diabetes*.

[30] Reichard P, Pihl M (1994). Mortality and treatment side-effects during long-term intensified conventional insulin treatment in the Stockholm Diabetes Intervention Study. *Diabetes*.

[31] UK Prospective Diabetes Study Group (UKPDS) (1998). Intensive blood-glucose control with sulphonylureas or insulin compared with conventional treatment and risk of complications in patients with type 2 diabetes (UKPDS 33). *Lancet*.

[32] Svendsen PA, Lauritzen T, Soegaard U, Nerup J (1982). Glycosylated haemoglobin and steady-state mean blood glucose concentration in type 1 (insulin-dependent) diabetes. *Diabetologia*.

[33] Santiago JV (1993). Lessons from the diabetes control and complications trial. *Diabetes*.

[34] The Diabetes Control and Complications Trial Research Group (1997). Hypoglycemia in the diabetes control and complications trial. *Diabetes*.

[35] Gold AE, Frier BM, MacLeod KM, Deary IJ (1997). A structural equation model for predictors of severe hypoglycaemia in patients with insulin-dependent diabetes mellitus. *Diabetic Medicine*.

[36] Cox DJ, Kovatchev BP, Julian DM (1994). Frequency of severe hypoglycemia in insulin-dependent diabetes mellitus can be predicted from self-monitoring blood glucose data. *Journal of Clinical Endocrinology and Metabolism*.

[37] Kovatchev BP, Cox DJ, Gonder-Frederick LA, Young-Hyman D, Schlundt D, Clarke W (1998). Assessment of risk for severe hypoglycemia among adults with IDDM: validation of the low blood glucose index. *Diabetes Care*.

[38] Kovatchev BP, Cox DJ, Kumar A, Gonder-Frederick L, Clarke WL (2003). Algorithmic evaluation of metabolic control and risk of severe hypoglycemia in type 1 and type 2 diabetes using self-monitoring blood glucose data. *Diabetes Technology and Therapeutics*.

[39] Cox DJ, Gonder-Frederick L, Ritterband L, Clarke W, Kovatchev BP (2007). Prediction of severe hypoglycemia. *Diabetes Care*.

[40] Amiel SA, Sherwin RS, Simonson DC, Tamborlane WV (1988). Effect of intensive insulin therapy on glycemic thresholds for counterregulatory hormone release. *Diabetes*.

[41] Amiel SA, Tamborlane WV, Simonson DC, Sherwin RS (1987). Defective glucose counterregulation after strict glycemic control of insulin-dependent diabetes mellitus. *New England Journal of Medicine*.

[42] Cryer PE, Gerich JE (1985). Glucose counterregulation, hypoglycemia, and intensive insulin therapy in diabetes mellitus. *New England Journal of Medicine*.

[43] White NH, Skor DA, Cryer PE (1983). Identification of type I diabetic patients at increased risk for hypoglycemia during intensive therapy. *New England Journal of Medicine*.

[44] Cryer PE (1992). Iatrogenic hypoglycemia as a cause of hypoglycemia-associated autonomic failure in IDDM: a vicious cycle. *Diabetes*.

[45] Henderson JN, Allen KV, Deary IJ, Frier BM (2003). Hypoglycaemia in insulin-treated Type 2 diabetes: frequency, symptoms and impaired awareness. *Diabetic Medicine*.

[46] Cryer PE (1997). *Hypoglycemia. Pathophysiology, Diagnosis and Treatment*.

[47] Cryer PE, Davis SN, Shamoon H (2003). Hypoglycemia in diabetes. *Diabetes Care*.

[48] Childs BP, Clark NG, Cox DJ (2005). Defining and reporting hypoglycemia in diabetes: a report from the American diabetes association workgroup on hypoglycemia. *Diabetes Care*.

[49] Cryer PE (2002). Hypoglycaemia: the limiting factor in the glycaemic management of Type I and Type II diabetes. *Diabetologia*.

[50] Cryer PE (1994). Hypoglycemia: the limiting factor in the management of IDDM. *Diabetes*.

[51] McCall AL, Kovatchev BP (2009). The median is not the only message: a clinician's perspective on mathematical analysis of glycemic variability and modeling in diabetes mellitus. *Journal of Diabetes Science and Technology*.

[52] Brownlee M, Hirsch IB (2006). Glycemic variability: a hemoglobin A1c-independent risk factor for diabetic complications. *Journal of the American Medical Association*.

[53] Hirsch IB, Brownlee M (2005). Should minimal blood glucose variability become the gold standard of glycemic control?. *Journal of Diabetes and Its Complications*.

[54] Esposito K, Giugliano D, Nappo F, Marfella R (2004). Regression of carotid atherosclerosis by control of postprandial hyperglycemia in type 2 diabetes mellitus. *Circulation*.

[55] Haffner S (2001). The importance of postprandial hyperglycaemia in development of cardiovascular disease in people with diabetes: point. *International Journal of Clinical Practice*.

[56] Monnier L, Mas E, Ginet C (2006). Activation of oxidative stress by acute glucose fluctuations compared with sustained chronic hyperglycemia in patients with type 2 diabetes. *Journal of the American Medical Association*.

[57] Temelkova-Kurktschiev TS, Koehler C, Henkel E, Leonhardt W, Fuecker K, Hanefeld M (2000). Postchallenge plasma glucose and glycemic spikes are more strongly associated with atherosclerosis than fasting glucose or HbA(1c) level. *Diabetes Care*.

[58] Clarke WL, Cox DJ, Gonder-Frederick L, Julian D, Kovatchev B, Young-Hyman D (1999). Biopsychobehavioral model of risk of severe hypoglycemia: self-management behaviors. *Diabetes Care*.

[59] Cox DJ, Gonder-Frederick LA, Kovatchev BP (1999). Biopsychobehavioral model of severe hypoglycemia II: understanding the risk of severe hypoglycemia. *Diabetes Care*.

[60] Gonder-Frederick L, Cox D, Kovatchev B, Schlundt D, Clarke W (1997). A biopsychobehavioral model of risk of severe hypoglycemia. *Diabetes Care*.

[61] Kovatchev B, Cox D, Gonder-Frederick L, Schlundt D, Clarke W (1998). Stochastic model of self-regulation decision making exemplified by decisions concerning hypoglycemia. *Health Psychology*.

[62] Nucci G, Cobelli C (2000). Models of subcutaneous insulin kinetics. A critical review. *Computer Methods and Programs in Biomedicine*.

[63] Wilinska ME, Chassin LJ, Schaller HC, Schaupp L, Pieber TR, Hovorka R (2005). Insulin kinetics in type-1 diabetes: continuous and bolus delivery of rapid acting insulin. *IEEE Transactions on Biomedical Engineering*.

[64] Bergman RN, Finegood DT, Ader M (1985). Assessment of insulin sensitivity in vivo. *Endocrine Reviews*.

[65] Bergman RN (2003). The minimal model of glucose regulation: a biography. *Advances in Experimental Medicine and Biology*.

[66] Bergman RN, Zaccaro DJ, Watanabe RM (2003). Minimal model-based insulin sensitivity has greater heritability and a different genetic basis than homeostasis model assessment or fasting insulin. *Diabetes*.

[67] Caumo A, Bergman RN, Cobelli C (2000). Insulin sensitivity from meal tolerance tests in normal subjects: a minimal model index. *Journal of Clinical Endocrinology and Metabolism*.

[68] Clausen JO, Borch-Johnsen K, Ibsen H (1996). Insulin sensitivity index, acute insulin response, and glucose effectiveness in a population-based sample of 380 young healthy Caucasians: analysis of the impact of gender, body fat, physical fitness, and life-style factors. *Journal of Clinical Investigation*.

[69] Ni TC, Ader M, Bergman RN (1997). Reassessment of glucose effectiveness and insulin sensitivity from minimal model analysis: a theoretical evaluation of the single-compartment glucose distribution assumption. *Diabetes*.

[70] Welch S, Gebhart SSP, Bergman RN, Phillips LS (1990). Minimal model analysis of intravenous glucose tolerance test-derived insulin sensitivity in diabetic subjects. *Journal of Clinical Endocrinology and Metabolism*.

[71] Dawson D, Vincent MA, Barrett EJ (2002). Capillary recruitment in skeletal muscle in response to exercise and hyperinsulinemia assessed with contrast-enhanced ultrasound. *American Journal of Physiology*.

[72] Mikines KJ, Sonne B, Farrell PA, Tronier B, Galbo H (1988). Effect of physical exercise on sensitivity and responsiveness to insulin in humans. *American Journal of Physiology*.

[73] Richter EA, Rowell LB, Shepherd JT (1996). Glucose utilization. *Handbook of Physiology*.

[74] Wasserman DH, Geer RJ, Rice DE (1991). Interaction of exercise and insulin action in humans. *American Journal of Physiology*.

[75] Pillonetto G, Caumo A, Sparacino G, Cobelli C (2006). A new dynamic index of insulin sensitivity. *IEEE Transactions on Biomedical Engineering*.

[76] Dalla Man C, Raimondo DM, Rizza RA, Cobelli C (2007). GIM, Simulation software of meal glucose-insulin model. *Journal of Diabetes Science and Technology*.

[77] Dalla Man C, Rizza RA, Cobelli C (2007). Meal simulation model of the glucose-insulin system. *IEEE Transactions on Biomedical Engineering*.

[78] Clarke WL, Cox D, Gonder-Frederick LA, Carter W, Pohl SL (1987). Evaluating clinical accuracy of systems for self-monitoring of blood glucose. *Diabetes Care*.

[79] The Diabetes Research In Children Network (Direcnet) Study Group (2003). A multicenter study of the accuracy of the one touch ultra home glucose meter in children with type 1 diabetes. *Diabetes Technology and Therapeutics*.

[80] Hirsch IB, Bode BW, Childs BP (2008). Self-monitoring of blood glucose (SMBG) in insulin- and non-insulin-using adults with diabetes: consensus recommendations for improving SMBG accuracy, utilization, and research. *Diabetes Technology and Therapeutics*.

[81] Freckmann G, Baumstark A, Jendrike N (2010). System accuracy evaluation of 27 blood glucose monitoring systems according to DIN en ISO 15197. *Diabetes Technology and Therapeutics*.

[82] Deiss D, Bolinder J, Riveline JP (2006). Improved glycemic control in poorly controlled patients with type 1 diabetes using real-time continuous glucose monitoring. *Diabetes Care*.

[83] Garg S, Zisser H, Schwartz S (2006). Improvement in glycemic excursions with a transcutaneous, real-time continuous glucose sensor: a randomized controlled trial. *Diabetes Care*.

[84] Kovatchev BP, Clarke WL (2007). Continuous glucose monitoring reduces risks for hypo- and hyperglycemia and glucose variability in diabetes. *Diabetes*.

[85] The Juvenile Diabetes Research Foundation Continuous Glucose Monitoring Study Group (2008). Continuous glucose monitoring and intensive treatment of type 1 diabetes. *New England Journal of Medicine*.

[86] Klonoff DC (2005). Continuous glucose monitoring: roadmap for 21st century diabetes therapy. *Diabetes Care*.

[87] Hovorka R (2006). Continuous glucose monitoring and closed-loop systems. *Diabetic Medicine*.

[88] Klonoff DC (2007). The artificial pancreas: how sweet engineering will solve bitter problems. *Journal of Diabetes Science and Technology*.

[89] Hirsch IB, Armstrong D, Bergenstal RM (2008). Clinical application of emerging sensor technologies in diabetes management: consensus guidelines for continuous glucose monitoring (CGM). *Diabetes Technology and Therapeutics*.

[90] Rebrin K, Steil GM, Van Antwerp WP, Mastrototaro JJ (1999). Subcutaneous glucose predicts plasma glucose independent of insulin: implications for continuous monitoring. *American Journal of Physiology*.

[91] Rebrin K, Steil GM (2000). Can interstitial glucose assessment replace blood glucose measurements?. *Diabetes Technology and Therapeutics*.

[92] Steil GM, Rebrin K, Hariri F (2005). Interstitial fluid glucose dynamics during insulin-induced hypoglycaemia. *Diabetologia*.

[93] Boyne MS, Silver DM, Kaplan J, Saudek CD (2003). Timing of changes in interstitial and venous blood glucose measured with a continuous subcutaneous glucose sensor. *Diabetes*.

[94] Kulcu E, Tamada JA, Reach G, Potts RO, Lesho MJ (2003). Physiological differences between interstitial glucose and blood glucose measured in human subjects. *Diabetes Care*.

[95] Stout PJ, Racchini JR, Hilgers ME (2004). A novel approach to mitigating the physiological lag between blood and interstitial fluid glucose measurements. *Diabetes Technology and Therapeutics*.

[96] Wentholt IME, Hart AAM, Hoekstra JBL, Devries JH (2007). Relationship between interstitial and blood glucose in type 1 diabetes patients: delay and the push-pull phenomenon revisited. *Diabetes Technology and Therapeutics*.

[97] Wientjes KJC, Schoonen AJM (2001). Determination of time delay between blood and interstitial adipose tissue glucose concentration change by microdialysis in healthy volunteers. *International Journal of Artificial Organs*.

[98] Aussedat B, Dupire-Angel M, Gifford R, Klein JC, Wilson GS, Reach G (2000). Interstitial glucose concentration and glycemia: implications for continuous subcutaneous glucose monitoring. *American Journal of Physiology*.

[99] Kovatchev BP, Clarke WL (2008). Peculiarities of the continuous glucose monitoring data stream and their impact on developing closed-loop control technology. *Journal of Diabetes Science and Technology*.

[100] Clarke WL, Kovatchev BP (2007). Continuous glucose sensors—continuing questions about clinical accuracy. *Journal of Diabetes Science and Technology*.

[101] The Diabetes Research in Children Network (DirecNet) Study Group (2008). The accuracy of the guardian RT continuous glucose monitor in children with type 1 diabetes. *Diabetes Technology and Therapeutics*.

[102] Kovatchev B, Anderson S, Heinemann L, Clarke W (2008). Comparison of the numerical and clinical accuracy of four continuous glucose monitors. *Diabetes Care*.

[103] Garg SK, Smith J, Beatson C, Lopez-Baca B, Voelmle M, Gottlieb PA (2009). Comparison of accuracy and safety of the SEVEN and the navigator continuous glucose monitoring systems. *Diabetes Technology and Therapeutics*.

[104] Heise T, Koschinsky T, Heinemann L, Lodwig V (2003). Hypoglycemia warning signal and glucose sensors: requirements and concepts. *Diabetes Technology and Therapeutics*.

[105] Bode B, Gross K, Rikalo N (2004). Alarms based on real-time sensor glucose values alert patients to hypo- and hyperglycemia: the Guardian continuous monitoring system. *Diabetes Technology and Therapeutics*.

[106] McGarraugh G, Bergenstal R (2009). Detection of hypoglycemia with continuous interstitial and traditional blood glucose monitoring using the FreeStyle navigator continuous glucose monitoring system. *Diabetes Technology and Therapeutics*.

[107] Noujaim SE, Horwitz D, Sharma M, Marhoul J (2007). Accuracy requirements for a hypoglycemia detector: an analytical model to evaluate the effects of bias, precision, and rate of glucose change. *Journal of Diabetes Science and Technology*.

[108] Ward WK (2004). The role of new technology in the early detection of hypoglycemia. *Diabetes Technology and Therapeutics*.

[109] Buckingham B, Cobry E, Clinton P (2009). Preventing hypoglycemia using predictive alarm algorithms and insulin pump suspension. *Diabetes Technology and Therapeutics*.

[110] Hughes CS, Patek SD, Breton MD, Kovatchev BP (2010). Hypoglycemia prevention via pump attenuation and red-yellow-green “traffic” lights using continuous glucose monitoring and insulin pump data. *Journal of Diabetes Science and Technology*.

[111] Kovatchev BP, Cox DJ, Gonder-Frederick LA, Clarke W (1997). Symmetrization of the blood glucose measurement scale and its applications. *Diabetes Care*.

[112] Service FJ, Molnar GD, Rosevear JW, Ackerman E, Gatewood LC, Taylor WF (1970). Mean amplitude of glycemic excursions, a measure of diabetic instability. *Diabetes*.

[113] Schlichtkrull J, Munck O, Jersild M (1965). The M-value, an index of blood glucose control in diabetics. *Acta Medica Scandinavica*.

[114] Ryan EA, Shandro T, Green K (2004). Assessment of the severity of hypoglycemia and glycemic lambility in type 1 diabetic subjects undergoing islet in transplantation. *Diabetes*.

[115] Kovatchev BP, Cox DJ, Gonder-Frederick L, Clarke WL (2002). Methods for quantifying self-monitoring blood glucose profiles exemplified by an examination of blood glucose patterns in patients with type 1 and type 2 diabetes. *Diabetes Technology and Therapeutics*.

[116] Kovatchev BP, Cox DJ, Farhy LS, Straume M, Gonder-Frederick L, Clarke WL (2000). Episodes of severe hypoglycemia in type 1 diabetes are preceded and followed within 48 hours by measurable disturbances in blood glucose. *Journal of Clinical Endocrinology and Metabolism*.

[117] Kovatchev BP, Straume M, Cox DJ, Farhy LS (2000). Risk analysis of blood glucose data: a quantitative approach to optimizing the control of Insulin Dependent Diabetes. *Journal of Theoretical Medicine*.

[118] Kovatchev BP, Otto E, Cox D, Gonder-Frederick L, Clarke W (2006). Evaluation of a new measure of blood glucose variability in diabetes. *Diabetes Care*.

[119] Kovatchev BP, Clarke WL, Breton M, Brayman K, McCall A (2005). Quantifying temporal glucose variability in diabetes via continuous glucose monitoring: mathematical methods and clinical application. *Diabetes Technology and Therapeutics*.

[120] Rodbard D (2009). New and improved methods to characterize glycemic variability using continuous glucose monitoring. *Diabetes Technology and Therapeutics*.

[121] Miller M, Strange P (2007). Use of Fourier models for analysis and interpretation of continuous glucose monitoring glucose profiles. *Journal of Diabetes Science and Technology*.

[122] Clarke W, Kovatchev B (2009). Statistical tools to analyze continuous glucose monitor data. *Diabetes Technology and Therapeutics*.

[123] Mcdonnell CM, Donath SM, Vidmar SI, Werther GA, Cameron FJ (2005). A novel approach to continuous glucose analysis utilizing glycemic variation. *Diabetes Technology and Therapeutics*.

[124] Sparacino G, Zanderigo F, Maran A, Cobelli C (2006). Continuous glucose monitoring and hypo/hyperglycaemia prediction. *Diabetes Research and Clinical Practice*.

[125] Sparacino G, Zanderigo F, Corazza G, Maran A, Facchinetti A, Cobelli C (2007). Glucose concentration can be predicted ahead in time from continuous glucose monitoring sensor time-series. *IEEE Transactions on Biomedical Engineering*.

[126] Reifman J, Rajaraman S, Gribok A, Ward WK (2007). Predictive monitoring for improved management of glucose levels. *Journal of Diabetes Science and Technology*.

[127] Zanderigo F, Sparacino G, Kovatchev B, Cobelli C (2007). Glucose prediction algorithms from continuous monitoring: assessment of accuracy via continuous glucose-error grid analysis. *Journal of Diabetes Science and Technology*.

[128] Cameron F, Niemayer G, Gundy-Burlet K, Buckingham B (2008). Statistical hypoglycemia prediction. *Journal of Diabetes Science and Technology*.

[129] Gani A, Gribok AV, Rajaraman S, Ward WK, Reifman J (2009). Predicting subcutaneous glucose concentration in humans: data-driven glucose modeling. *IEEE Transactions on Biomedical Engineering*.

[130] Eren-Oruklu M, Cinar A, Quinn L, Smith D (2009). Estimation of future glucose concentrations with subject-specific recursive linear models. *Diabetes Technology and Therapeutics*.

[131] Pappada SM, Cameron BD, Rosman PM (2008). Development of a neural network for prediction of glucose concentration in type 1 diabetes patients. *Journal of Diabetes Science and Technology*.

[132] Cobelli C, Dalla Man C, Sparacino G, Magni L, Nicolao G, Kovatchev BP (2009). Diabetes: models, signals, and control. *IEEE Reviews in Biomedical Engineering*.

[133] LeBlanc H, Chauvet D, Lombrail P, Robert JJ (1986). Glycemic control with closed-loop intraperitoneal insulin in type I diabetes. *Diabetes Care*.

[134] Saudek CD, Selman JL, Pitt HA (1989). A preliminary trial of the programmable implantable medication system for insulin delivery. *New England Journal of Medicine*.

[135] Broussolle C, Jeandidier N, Hanaire-Broutin H (1994). French multicentre experience of implantable insulin pumps. *Lancet*.

[136] Hanaire-Broutin H, Broussolle C, Jeandidier N (1995). Feasibility of intraperitoneal insulin therapy with programmable implantable pumps in IDDM: a multicenter study. *Diabetes Care*.

[137] Oskarsson PR, Lins PE, Henriksson HW, Adamson UC (1999). Metabolic and hormonal responses to exercise in type 1 diabetic patients during continuous subcutaneous, as compared to continuous intraperitoneal, insulin infusion. *Diabetes and Metabolism*.

[138] Oskarsson PR, Lins PE, Backman L, Adamson UC (2000). Continuous intraperitoneal insulin infusion partly restores the glucagon response to hypoglycaemia in type 1 diabetic patients. *Diabetes and Metabolism*.

[139] Catargi B, Meyer L, Melki V, Renard E, Jeandidier N (2002). Comparison of blood glucose stability and HbA1C between implantable insulin pumps using U400 hoe 21pH insulin and external pumps using lispro in type 1 diabetic patients: a pilot study. *Diabetes and Metabolism*.

[140] Renard E (2002). Implantable closed-loop glucose-sensing and insulin delivery: the future for insulin pump therapy. *Current Opinion in Pharmacology*.

[141] Renard E, Costalat G, Chevassus H, Bringer J (2006). Artificial *β*-cell: clinical experience toward an implantable closed-loop insulin delivery system. *Diabetes and Metabolism*.

[142] Renard E, Place J, Cantwell M, Chevassus H, Palerm CC (2010). Closed-loop insulin delivery using a subcutaneous glucose sensor and intraperitoneal insulin delivery: feasibility study testing a new model for the artificial pancreas. *Diabetes Care*.

[143] Bellazzi R, Nucci G, Cobelli C (2001). The subcutaneous route to insulin-dependent diabetes therapy: closed-loop and partially closed-loop control strategies for insulin delivery and measuring glucose concentration. *IEEE Engineering in Medicine and Biology Magazine*.

[144] Hovorka R, Chassin LJ, Wilinska ME (2004). Closing the loop: the adicol experience. *Diabetes Technology and Therapeutics*.

[145] Hovorka R, Canonico V, Chassin LJ (2004). Nonlinear model predictive control of glucose concentration in subjects with type 1 diabetes. *Physiological Measurement*.

[146] Steil GM, Rebrin K, Darwin C, Hariri F, Saad MF (2006). Feasibility of automating insulin delivery for the treatment of type 1 diabetes. *Diabetes*.

[148] Clarke WL, Kovatchev B (2007). The artificial pancreas: how close are we to closing the loop?. *Pediatric Endocrinology Reviews*.

[149] Cobelli C, Renard E, Kovatchev BP (2011). Perspectives in diabetes: artificial pancreas: past, present, future. *Diabetes*.

[150] Kovatchev BP, Breton MD, Dalla Man C, Cobelli C (2009). In silico preclinical trials: a proof of concept in closed-loop control of type 1 diabetes. *Journal of Diabetes Science and Technology*.

[151] Kovatchev B, Cobelli C, Renard E (2010). Multinational study of subcutaneous model-predictive closed-loop control in type 1 diabetes mellitus: summary of the results. *Journal of Diabetes Science and Technology*.

[152] Zisser H, Jovanovic L, Doyle F, Ospina P, Owens C (2005). Run-to-run control of meal-related insulin dosing. *Diabetes Technology and Therapeutics*.

[153] Owens C, Zisser H, Jovanovic L, Srinivasan B, Bonvin D, Doyle FJ (2006). Run-to-run control of blood glucose concentrations for people with type 1 diabetes mellitus. *IEEE Transactions on Biomedical Engineering*.

[154] Palerm CC, Zisser H, Bevier WC, Jovanovič L, Doyle FJ (2007). Prandial insulin dosing using run-to-run control: application of clinical data and medical expertise to define a suitable performance metric. *Diabetes Care*.

[155] Magni L, Raimondo F, Bossi L (2007). Model predictive control of type 1 diabetes: an in silico trial. *Journal of Diabetes Science and Technology*.

[156] Weinzimer SA, Steil GM, Swan KL, Dziura J, Kurtz N, Tamborlane WV (2008). Fully automated closed-loop insulin delivery versus semiautomated hybrid control in pediatric patients with type 1 diabetes using an artificial pancreas. *Diabetes Care*.

[157] Clarke WL, Anderson S, Breton M, Patek S, Kashmer L, Kovatchev B (2009). Closed-loop artificial pancreas using subcutaneous glucose sensing and insulin delivery and a model predictive control algorithm: the Virginia experience. *Journal of Diabetes Science and Technology*.

[158] Bruttomesso D, Farret A, Costa S (2009). Closed-loop artificial pancreas using subcutaneous glucose sensing and insulin delivery and a model predictive control algorithm: preliminary studies in Padova and Montpellier. *Journal of Diabetes Science and Technology*.

[159] Hovorka R, Allen JM, Elleri D (2010). Manual closed-loop insulin delivery in children and adolescents with type 1 diabetes: a phase 2 randomised crossover trial. *The Lancet*.

[160] El-khatib FH, Russell SJ, Nathan DM, Sutherlin RG, Damiano ER (2010). A bihormonal closed-loop artificial pancreas for type 1 diabetes. *Science Translational Medicine*.

[161] Dassau E, Zisser H, Palerm CC, Buckingham BA, Jovanovič L, Doye FJ (2008). Modular artificial *β*-cell system: a prototype for clinical research. *Journal of Diabetes Science and Technology*.

[162] Kovatchev B, Patek S, Dassau E (2009). Control to range for diabetes: functionality and modular architecture. *Journal of Diabetes Science and Rechnology*.

[163] Atlas E, Nimri R, Miller S, Grunberg EA, Phillip M (2010). MD-logic artificial pancreas system: a pilot study in adults with type 1 diabetes. *Diabetes Care*.

[164] Dassau E, Zisser H, Percival MW, Grosman B, Jovanovič L, Doyle FJ Clinical results of automated artificial pancreatic *β*-cell system with unannounced meal using multi-parametric MPC and insulin-on-board.

[165] Renard EM, Farret A, Place J, Cobelli C, Kovatchev BP, Breton MD (2010). Closed-loop insulin delivery using subcutaneous infusion and glucose sensing, and equipped with a dedicated safety supervision algorithm, improves safety of glucose control in type 1 diabetes. *Diabetologia*.

[166] Hovorka R, Kumareswaran K, Harris J (2011). Overnight closed loop insulin delivery (artificial pancreas) in adults with type 1 diabetes: crossover randomized controlled studies. *British Medical Journal*.

[167] Zisser H, Dassau E, Bevier W Initial evaluation of a fully automated artificial pancreas.

[168] Breton MD, Farret A, Bruttomesso D (2012). Fully-integrated artificial pancreas in type 1 diabetes: modular closed-loop glucose control maintains near-normoglycemia. *Diabetes*.

[169] Cobelli C, Renard E, Kovatchev  BP (2012). Pilot studies of wearable artificial pancreas in type 1 diabetes. *Diabetes Care*.

